# A review of recent advances in exosomes and allergic rhinitis

**DOI:** 10.3389/fphar.2022.1096984

**Published:** 2022-12-15

**Authors:** Zhong Zheng, Yangyang Yu

**Affiliations:** ^1^ Department of Child Otorhinolaryngology, Anhui Provincial Children’s Hospital, Hefei, China; ^2^ Department of Function Examination Center, Anhui Chest Hospital, Hefei, China

**Keywords:** exosome, allergic rhinitis, mechanism, miRNA, lncRNA, treatment

## Abstract

Allergic rhinitis is a chronic inflammatory disease of nasal mucosa caused by the presence of IgE after exposure to allergens, characterized by nasal irritation, hypersecretion of the nasal passages and sneezing, which frequently occurs in children and adolescents. There has been an increase in allergic rhinitis over the past few years due to air pollution. Exosomes have been discovered to be nano-sized vesicles, which contain a wide range of substances, including proteins and nucleic acids, numerous studies indicates that exosomes play a vital role in cells communication. Recently there have been more and more studies exploring the role of exosomes in allergic rhinitis. Therefore, here we will present a comprehensive review of the research on exosomes and their role in allergic rhinitis for the purpose of providing new understanding of potential value of exosomes applied to the treatment of allergic rhinitis.

## Introduction

Allergic rhinitis is a common disease, which is becoming an increasingly serious global problem related to health, medicine, and economics. High-income countries show a prevalence of up to 50%, making it one of the most common chronic conditions, while the prevalence is comparatively low in low- and middle-income countries, despite the fact that it is steadily rising in these nations (Bousquet et al., 2020; Liu and Liu, 2022). In Europe, over the last 3 decades, there has been a gradual increase from 19% to 32% of Danish adults suffering from allergic rhinitis (Leth-Moller et al., 2020). Its high incidence imposes a substantial burden on our general welfare, as well as significant financial costs, both direct and indirect. In allergic rhinitis, inhaled particles cause an inflammatory response led by IgE, which may result in sneezing, nasal itching, rhinorrhea, or nasal obstruction, among other symptoms (Mims, 2014). A number of allergens can trigger allergic rhinitis, including pollen (from trees, grass, and weeds), mould and dust mites et al. During the year, allergens are classified into perennial and seasonal triggers according to their temporal pattern. In some cases, perennial symptoms occur in patients because of things in their homes all year round, it may include mold, dust mites, or animals (especially cats and dogs) (Schuler Iv and Montejo, 2019). Lifestyle factors and climate change contribute to high prevalence of allergic rhinitis, including antibiotic use, contact with farm animals, exposure to air pollution, and parental smoking et al. It is noteworthy that genetic and environmental factors also contribute to other allergic diseases, such as atopic dermatitis and asthma, which are often comorbid with allergic rhinitis (2020; Zhang et al., 2021). In terms of treating allergic rhinitis, there are three main approaches. These approaches are avoidance, medications, and immunotherapy (Schuler Iv and Montejo, 2021). However, allergic rhinitis cannot be cured completely at present, and can only be prevented and controlled through standardized comprehensive treatment. There is a mental health impact associated with allergic rhinitis as well as physical health effects (Amritwar et al., 2017). Due to its high prevalence and the difficulty in treating allergic rhinitis, the exploration of active and effective diagnoses and treatments is essential.

Exosomes are lipid bilayer vesicles released *via* exocytosis, an exocytotic process, with a diameter of 40–100 nm. A wide variety of bioactive molecules can be found in exosomes, including proteins, lipids, DNA, and microRNA. There is evidence of its presence in various body fluids, including urine, blood, breast milk, saliva, cerebrospinal fluid and amniotic fluid (Konala et al., 2016; Pegtel and Gould, 2019; Kalluri and LeBleu, 2020). Recently researchers have found that exosomes may be involved in occurrence and progression of a variety of diseases, such as tumors (Zhang and Yu, 2019), neurodegenerative diseases (Jiang et al., 2019; Wang et al., 2020), infection (Choi et al., 2020; Ocansey et al., 2020), autoimmune diseases (Robak et al., 1998; Lee et al., 2016) and cardiovascular disorders (Zamani et al., 2019) et al., among them allergic rhinitis is a newly studied exosome related disease. Currently, research on exosomes and allergic rhinitis has gained increasing attention among scholars. As a result, here we will present a comprehensive review of the research on exosomes in order to understand how exosomes might be useful in treating allergic rhinitis.

### Allergic rhinitis pathogenesis

It remains to be explored in greater depth about the mechanisms underlying allergic rhinitis pathology, despite extensive studies of the mechanisms that contribute to the disease. Allergic rhinitis is a result of specific IgE-mediated responses to allergens inhaled, which is leaded by T helper two cells (Th2). Eosinophils and basophils are influxed into the mucosa when allergic rhinitis causes mucosal inflammation (Bernstein et al., 2016). IgE is provided by B cells, T cells and basophils et al. in conjunction with each other, which is also involved in multiple cytokines. An understanding of allergic rhinitis pathogenesis may lead to better treatment. Therefore, this review summarizes recent studies that illustrate the role of important cells and proteins in allergic rhinitis pathogenesis, to provide support for relevant research. Numerous studies indicate that several cell types play a role in allergic rhinitis’ occurrence and progression. These cells consist mainly of epithelial cells, dendritic cells (DCs), type 2 follicular helper T cells (Tfh2), basophils, mast cells, Th2 cells, B cells, group 2 innate lymphoid cells (ILC2s), eosinophils and neutrophils (Drazdauskaite et al., 2020; Zhang et al., 2022). Among these cells, the ILC2s play a crucial role in airway inflammation as a component of the Th2 innate immunity. In fact, within an ILC2-mediated immune microenvironment, mast cells, histiocytes and Th2 cells can produce IL-4, IL-5, IL-9, IL-13, IL-25, and IL-33 (Peng et al., 2020). In addition, many proteins contribute to the occurence and progression of allergic rhinitis. Toll-like receptor 4 (TLR4) binds to the corresponding ligands (HMGB1 and LPS et al.) to activate the relevant signaling pathway, resulting to the activation and translocation of NF-κB, which induces allergic rhinitis by mediating cytokine secretion (Sha et al., 2008; Lauriello et al., 2012; Cui et al., 2015; Radman et al., 2015; Haruna et al., 2019). YKL-40 (chitinase-3-like-1; CHI3L1) is primarily known as human cartilage glycoprotein 39 (HCgp-39), which is an important protein that plays a crucial role in chronic hypersensitivity inflammation. A high level of YKL-40 expression is found in nasal mucosa of patients with mild and moderate/severe allergic rhinitis (Pirayesh et al., 2020), which participates in mucosal remodeling in the nasal cavity, mediates the epithelial detachment of nasal mucosa, tissue edema and small vessel hyperplasia, and aggravating the symptoms of allergic rhinitis (Sanai et al., 1999; Kim et al., 2019). YKL-40 may promote nasal mucosa remodeling by activating fibroblasts and producing TIMP1 and MMP-9 (Park et al., 2013). Histone deacetylase (HDAC) is an important driver of inflammation in response to allergies, as well as tight junction dysfunction, which may be a pathogenesis of airway epithelial tissue and cell damage, and that inhibiting HDAC may restore epithelial barrier integrity (Steelant et al., 2019). There is also evidence that periostin may be important in allergic rhinitis. It has been suggested that it transmits signals to trigger allergic diseases while secreting mucus (Izuhara et al., 2016; Izuhara et al., 2019). Moreover, the CD86 may function as a second activation signal for T cells, make the initial T lymphocytes differentiate to Th2 cells, lead to the imbalance of Th1/Th2 cytokines in the body, and mediate the occurrence of allergic rhinitis (Gunawan et al., 2015; Li et al., 2016b; Sun et al., 2019).

### Exosomes in allergic diseases

The exosome is a type of small extracellular vesicle secreted by cells that contains protein, lipid, and nucleic acid for physiological and pathological processes. According to cellular biogenesis and sizes, they can be divided into three groups to deliver their contents and enable the exchange of information between cells, including docking and fusing with the recipient cell plasma membrane, targeting the recipient’s signals, and internalizing into the recipient (Konala et al., 2016; Kalluri and LeBleu, 2020; Zhang et al., 2020). Exosomes are found in a variety of body fluids, which can be produced by almost all types of normal cells, including mesenchymal stem cells, T cells, B cells, and macrophages et al. (Cheng et al., 2017; He et al., 2020). Exosomes were originally regarded as molecular ‘garbage bags’ associated with cell waste disposal (Harischandra et al., 2019). Nevertheless, recently they have become crucial devices for intercellular communication, modulating and mediating various cellular functions. (Shekari et al., 2021). Studies have shown that RNA (including mRNAs, miRNAs, and other non-coding RNAs), DNA, and lipids can be incorporated into intraluminal vesicles from exosomes actively and selectively (van Niel et al., 2018). According to the ExoCarta database (http://www.exocarta.org/), 9769 proteins, 3408 mRNAs, 2838 miRNAs, and 116 lipids have been discovered in exosomes as of 2020 (Zhou et al., 2020). Almost all cells can communicate from cell to cell by secreting exosomes, which are closely related to their physiological effects (Mathieu et al., 2019). Research has shown that exosomes are important in treating inflammatory diseases (Harrell et al., 2019; Zhao and Luo, 2019; Choi et al., 2020; Ocansey et al., 2020), neurological diseases (Jiang et al., 2019; Wang et al., 2020; Harrell et al., 2021) and autoimmune diseases (Robak et al., 1998; Lee et al., 2016; Riazifar et al., 2019). Furthermore, exosomes have been identified as tumor markers and a diagnostic tool (Sharma and Johnson, 2020; Li et al., 2021). This review focuses primarily on recent research progress related to exosomes in allergies. As a result of exposure to allergens, the body’s initial T cells differentiate into Th2 cells, which produce cytokines that promote allergic reactions. A study has shown that exosomes secreted by T cells reduce the production of inflammatory mediators when worms cause host allergic reactions. Additionally, exosomes secreted by worms contain miRNA, which can enter immune cells and promote their proliferation and differentiation (Siles-Lucas et al., 2015). Moreover, Li et al. found that exosomes secreted by bone marrow mast cells (BMMCs) promoted the proliferation of naive CD4^+^ T cells, and significantly enhanced Th2 cell differentiation by ligation of OX40L and OX40 between BMMC-exosomes and CD4^+^ T cells and this may represent a novel mechanism for communicating between cells (Li et al., 2016a). Interestingly, Gao et al. discovered that exosomes from septic mice have been shown to enhance differentiation of Th1 and Th2 cells and promote proliferation and migration of T cells for the first time (Gao et al., 2019). Generally, studies on exosomes in allergic reactions mainly focus on T cells and mast cells. Exosomes may play a significant role in activating or suppressing immune cells in this process. In addition, there is something worth mentioning that for allergy, Villalba et al. (2014) have demonstrated that exosomes derived from bronchoalveolar lavage of Ole e 1-tolerized mice were effective in protecting animals against allergic sensitization to Ole e 1 and the non-related allergen Bet v 1, as opposed to naive mice’s exosomes, this suggested that exosomes could be a better allergy vaccine due to their properties.

### Overview of research related to allergic rhinitis and exosomes

A growing body of researches has been conducted on exosomes and allergic rhinitis in recent years. It has been demonstrated that exosomes play a vital role in allergic rhinitis’ occurrence and progression. In this section, we review the relevant studies regarding exosomes and allergic rhinitis to clarify the corresponding mechanisms and provide theoretical support for targeting exosomes therapy of allergic rhinitis.

**FIGURE 1:**
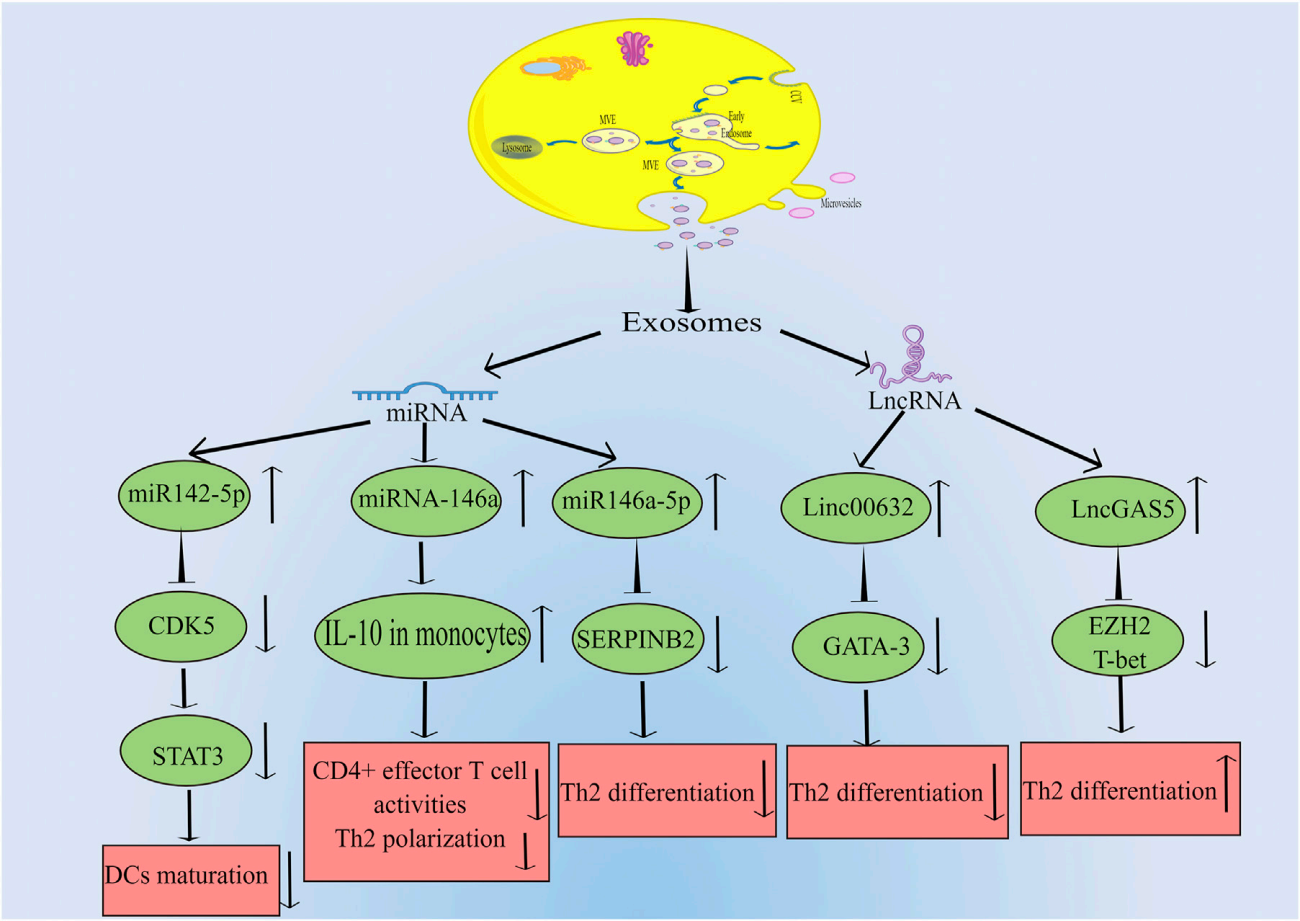
Exosomes can be produced by many kinds of cells, which can transmit intercellular signals through the release of miRNA and LncRNA, and affect the expression of related genes, thus leading to the occurrence and progression of allergic rhinitis. The main miRNAs released by exosomes are miR142-5p, miR146a and miR146-5p. MiR142-5p can inhibit the expression of CDK5, which leads to the decrease of STAT3 and ultimately inhibits DCs maturation. MiR146a can increase the level of IL-10 in monocytes, and then inhibit CD4^+^ effector T cell activity and Th2 polarization. MiR146-5p can inhibit the expression of SERPINB2 and lead to the decrease of Th2 differentiation. LncRNAs mainly include Linc00632 and LncGAS5. Linc00632 can inhibit the expression of GATA-3 and lead to the decrease of Th2 differentiation. While LncGAS5 can inhibit the expression of EZH2 and T-bet, ultimately increases Th2 differentiation (By Figdraw).

In a study by Qiu et al., they found that exosomes can be discovered in the patients with chronic atypical allergic rhinitis, and these exosomes containing microbial products and airborne antigens, which can influence DCs maturation and major histocompatibility class I (MHCI) production, thereby promoting antigen specific CD8^+^ T cell development, eventually this leads to allergic rhinitis (Qiu et al., 2011; Qiu et al., 2012). In another study by Wu et al. (2015) they found that in nasal mucus from allergic rhinitis patients, 21 vesicle miRNAs were up-regulated and 14 were down-regulated significantly compared to healthy controls. And the studied vesicles were confirmed to be exosomes by FACS analysis and binding specifically to antiCD63 coated latex beads. By bioinformatic analysis, this study demonstrated that vesicle miRNA may be a regulator for the development of allergic rhinitis. Interestingly, Jiang et al. (2022) indicated that there are 812 miRNAs were detected in the serum exosomes, including 16 upregulated and 14 downregulated ones. Their study also suggested that children suffering from allergic rhinitis were predicted to respond to SCIT by serum exosomal hsa-miR-4669. Actually, there are many studies on miRNA secreted by exosome and allergic rhinitis. For example, Luo et al. (2015) found that patients with allergic rhinitis had much lower miR146a levels than healthy subjects in nasal epithelial specimens. And there is evidence that human nasal epithelial cells produce miR-146a, which can be released to the microenvironment carried by exosomes. Their results indicated that miRNA-146a can inhibit CD4^+^ effector T cell activity and Th2 polarization by enhancing monocyte interleukin-10 expression and the allergic reaction was suppressed in the mouse nasal mucosa ultimately. Furthermore, Zhou et al. (2021) discovered that there was a significant increase in Th2 cells in allergic rhinitis patients compared to healthy donors, while exosomes from HMSCs could reduce the expression of SERPINB2 and facilitate the differentiation of Th2 cells. They concluded that the miR-146a-5p and SERPINB2 genes can serve as potential targets for allergic rhinitis therapy . Additionally, it is acknowleged that exosomes can influence the DCs maturation. Teng et al. used RNA-seq and results showed that miR-142-5p was the differentially decreased gene in Tfh-derived exosomes. In depth, they indicated that miR-142-5p inhibited DCs maturation by inhibiting CDK5 and STAT3 expression. According to their findings, Tfh-derived exosomes play a central role in allergic rhinitis pathogenesis by regulating the miR-142-5p/CDK5/STAT3 signaling pathway axis (Teng et al., 2022). It has been discussed above how miRNA secreted by exosomes influences the pathogenesis of allergic rhinitis. Interestingly, exosomes can also release long noncoding RNA (LncRNA), thus affecting the occurrence and progression of allergic rhinitis. Zhu et al. (2020) found that the expression of LncGAS5 was up-regulated in exosomes secreted by nasal epithelial cells of patients with allergic rhinitis, while LncGAS5 can promote the differentiation of Th2 cells by inhibiting the expression of transcription factor T-bet and EZH2. Their results suggested that LncGAS5 in allergic rhinitis exosomes plays a critical role in Th1/Th2 differentiation, providing a potential therapeutic target. Moreover, Li et al. (2022) showed that there was a significant drop in Linc00632 expression in nasal mucosa of allergic rhinitis patients by four times. Exosomes derived from human umbilical cord mesenchymal stem cells showed dramatic inhibition of Th2 differentiation, inhibited GATA binding protein-3 (GATA-3) expression, and decreased IL-4 levels in CD4^+^ T cells. Further research revealed that the interaction between Linc00632 and EZH2 inhibited the expression of GATA-3. As discussed above, current studies on exosome-mediated allergic rhinitis pathogenesis are mainly conducted through miRNA and LncRNA secreted by exosomes from different cell sources. The relevant signal regulation is shown in Figure 1.

## Conclusion

It is acknowleged that allergy rhinitis is a pathological condition in which exosomes act as inflammatory mediators, promote or inhibit cell proliferation and differentiation, and present antigens. There is a possibility that further research could lead to the development of nasal sprays delivering therapeutic drugs or genes to the nasal mucosa in allergy rhinitis using exosomes. For example, it may be possible to induce tolerance by nasal vaccines based on exosomes *via* intranasal administration. With allergy vaccines containing allergen-modified molecules, new delivery systems, and alternative routes of administration, allergy patients’ daily lives will be improved as well as unraveling the immunological mechanisms that underlie immunotherapy in order to develop new therapeutic approaches (Villalba et al., 2014). However, there are currently few studies on the effect of exosomes in allergic rhinitis pathogenesis. In addition to other extracellular vesicles, it remains unclear whether exogenous drugs or genes will have additional undiscovered side effects. It is also important to support more relevant experiments and to simplify exosome preservation conditions. Extra studies are therefore indispensable to determine how exosomes are formed, released, and transported as well as their role in allergic rhinitis. A potential focus of allergic rhinitis research may be to understand the functions of exosomes in regulating mast cells and mucin-secreting epithelial tissues. Meanwhile, exosomes produced by mesenchymal stem cells (MSCs) may prove potent in treating allergic rhinitis for their immunosuppressive properties, tissue repair ability, and secretion of various biological factors. Actually, numerous clinical and preclinical studies have proven the efficacy of MSC-based therapy for a number of allergic diseases, and the mechanisms related to these interventions have been explored (Li et al., 2020). Overall, targeted exosomes is a very promising treatment for allergic rhinitis that is expected to require many participants in the future(Shen et al., 2018).
